# A Dynamic Prediction Model for Renal Progression in Primary Membranous Nephropathy

**DOI:** 10.7150/ijms.95321

**Published:** 2024-05-13

**Authors:** Yufeng Liang, Qiu Li, Zhenhuan Zou, Binsan Huang, Nan Zhong, Chenlun Li, Azhen Wang, Yongping Chen, Shuzhen Tu, Jianxin Wan

**Affiliations:** 1 Department of Nephrology, Blood Purification Research Center, The First Affiliated Hospital, Fujian Medical University, Fuzhou 350005, China.; 2 Department of Nephrology, The Second Hospital of Longyan, Fujian 364000, China.; 3 Fujian Clinical Research Center for Metabolic Chronic Kidney Disease, The First Affiliated Hospital, Fujian Medical University, Fuzhou 350005, China.; 4 Department of Nephrology, National Regional Medical Center, Binhai Campus of the First Affiliated Hospital, Fujian Medical University, Fuzhou 350005, China.; 5 Department of Orthopedics, The Second Hospital of Longyan, Fujian 364000, China.; 6 School of Mathematics and Information Engineering, Longyan University, Fujian, 364000, China.

**Keywords:** web-based, dynamic, prediction model, nomogram, primary membranous nephropathy, renal progression.

## Abstract

**Objective:** This study aimed to build and validate a practical web-based dynamic prediction model for predicting renal progression in patients with primary membranous nephropathy (PMN).

**Method:** A total of 359 PMN patients from The First Affiliated Hospital of Fujian Medical University and 102 patients with PMN from The Second Hospital of Longyan between January 2018 to December 2023 were included in the derivation and validation cohorts, respectively. Renal progression was delineated as a decrease in eGFR of 30% or more from the baseline measurement at biopsy or the onset of End-Stage Renal Disease (ESRD). Multivariable Cox regression analysis was employed to identify independent prognostic factors. A web-based dynamic prediction model for renal progression was built and validated, and the performance was assessed using. An analysis of the receiver operating characteristic and the decision curve analysis.

**Results:** In the derivation cohort, 66 (18.3%) patients experienced renal progression during the follow-up period (37.60 ± 7.95 months). The final prediction rule for renal progression included hyperuricemia (HR=2.20, 95%CI 1.26 to 3.86), proteinuria (HR=2.16, 95%CI 1.47 to 3.18), significantly lower serum albumin (HR=2.34, 95%CI 1.51 to 3.68) and eGFR (HR=1.96, 95%CI 1.47 to 2.61), older age (HR=1.85, 95%CI 1.28 to 2.61), and higher sPLA2R-ab levels (HR=2.08, 95%CI 1.43 to 3.18). Scores for each variable were calculated using the regression coefficients in the Cox model. The developed web-based dynamic prediction model, available online at http://imnpredictmodel1.shinyapps.io/dynnomapp, showed good discrimination (C-statistic = 0.72) and calibration (Brier score, P = 0.155) in the validation cohort.

**Conclusion:** We developed a web-based dynamic prediction model that can predict renal progression in patients with PMN. It may serve as a helpful tool for clinicians to identify high-risk PMN patients and tailor appropriate treatment and surveillance strategies.

## 1. Introduction

Primary membranous nephropathy (PMN) is a progressive disease that can lead to various outcomes, including spontaneous remission and end-stage renal disease (ESRD) 1, 2. When untreated, approximately 60% of patients with PMN may experience a decline in renal function. However, immunosuppressive therapies, while improving outcomes, may also increase potential toxicities and additional financial burdens. Appropriate surveillance and precise intervention may alter the trajectory of PMN and possibly improve patient prognosis. Therefore, it is crucial to predict the risk of renal progression to guide treatment strategies and avoid unnecessary therapy.

Several factors have been identified to be associated with renal prognosis in patients with PMN, such as age 3, proteinuria 4, 5, pathologic severity 6, 7, race, smoking 8, hypercholesterolemia 9, hyperuricemia 9, hyperglycemia 10, and hypertension 7. Risk stratification models based on readily available clinical characteristics are increasingly utilized to aid in early intervention strategies for PMN. Some renal progression prediction models factor in estimated glomerular filtration rate (GFR) and proteinuria 11-13. Recently, serum M-type phospholipase A2 receptor autoantibodies (sPLA2R-ab) have emerged as a landmark in estimating renal prognosis 1, 14. Currently, there are some established predictive models, including one that incorporates sPLA2R-ab 11, 13, 15. However, there are variations in prognoses among patients with the same rating in existing scoring systems 11, 13, 15, necessitating the inclusion of additional variables to construct a new model. Therefore, we enrolled an extended Chinese PMN cohort to develop an updated prediction model for the prediction of renal progression in PMN. The purpose of this study is to develop a user-friendly web-based clinical prediction model to estimate the conditional risk of patient-specific renal progression and externally validate it to guide individual treatment decision-making.

## 2. Materials and Methods

### 2.1 The study population

A total of 376 patients with biopsy-diagnosed PMN between January 2018 and December 2023 in The First Affiliated Hospital of Fujian Medical University (Fujian, China) were included in the derivation cohort. The inclusion criteria were individuals aged ≥15 years with a diagnosis of PMN confirmed by kidney biopsy. The exclusion criteria included: (1) membranous nephropathy secondary to such conditions as autoimmune disease, malignancy, and hepatitis B; (2) patients received immunosuppressive therapy or renal replacement treatment before hospitalization; (3) severe heart failure or hepatic failure 11. Ultimately, 359 patients were eligible for this analysis in the derivation cohort. All patients provided written informed consent.

For the validation cohort, 108 patients with biopsy-diagnosed PMN between January 2018 to December 2023 at The Second Hospital of Longyan (Fujian, China) were included. All patients were followed up for at least one year. After excluding 6 patients, 102 patients were included in the validation cohort.

### 2.2 Clinical measures

The estimated glomerular filtration rate (eGFR) was calculated using the Chronic Kidney Disease Epidemiology Collaboration (CKD-EPI) formula. The levels of sPLA2Rab were measured by an enzyme-linked immunosorbent assay (ELISA, Euroimmun AG, Lubeck, Germany) test at biopsy, and sPLA2R-Ab levels ≥ 20 RU/mL were considered positive. Similar to a previous study 14, sPLA2R-Ab levels were divided into low, medium, and high titer groups with titer < 100 RU/mL, 100-320 RU/mL, and > 320 RU/mL. Patients were followed up until the endpoint was reached or December 2023.

### 2.3 Treatment strategy

The treatment strategies were determined by nephrologists in accordance with the Kidney Disease: Improving Global Outcomes (KDIGO) guidelines 16. Almost all patients received angiotensin-converting enzyme inhibitors (ACEIs) or angiotensin receptor blockers (ARBs)therapy. Patients were stratified into five groups based on their immunosuppressive (IS) treatment regimens: 1) None, no immunosuppressants; 2) Cyclophosphamide (CTX), receiving corticosteroids and CTX; 3) Calcineurin inhibitor (CNI), receiving corticosteroids and tacrolimus or cyclosporine; 4) RTX, receiving rituximab; 5) Other, receiving a combination of immunosuppressants or treatments not included in the above categories, such as monotherapy with Tripterygium wilfordii polyglycoside.

### 2.4 Pathological measures

Renal biopsies were assessed and scored by two experienced nephropathologists. The diagnosis of PMN was established based on pathological parameters, including Ehrenreich-Churg stage, glomerulosclerosis, interstitial fibrosis/tubular atrophy, crescent formation, and immunohistological staining (IgG subgroup, IgA, IgM, C3, and C1q). Renal PLA2R antigen was detected in renal biopsy specimens 1, 14, 17.

### 2.5 Renal outcome

The endpoint of this study was renal function progression, defined by a reduction in eGFR greater than or equal to 30% compared with baseline renal function at biopsy 13, ESRD, which was defined as the initiation for dialysis or renal transplant. Treatment response: Treatment response can be classified into remission and non-remission. Remission is defined as achieving clinical remission (complete remission and partial remission) after treatment, which includes complete remission (proteinuria < 0.3 g/day) and partial remission (proteinuria < 3.5 g/day but ≥ 0.3 g/day). Non-remission refers to the failure to achieve clinical remission after treatment, or the occurrence of poor prognostic events such as doubling of serum creatinine or relapse 6, 16. The renal outcomes were evaluated by the review of medical records or telephone communication in the clinics or hospitals.

### 2.6 Date collection

The patient's clinical and serum laboratory data were collected from medical records at the time of biopsy. The eGFR data were checked by two authors (Liang YF and Zhou ZH) without other medication use.

### 2.7 Statistical analyses

All statistical analyses were performed with IBM SPSS software (version 20.0, SPSS Inc., Chicago, IL, USA) and R software (version 3.4.3). The association of variables with renal progression was assessed using Cox proportional hazards models. Risk factors for renal progression were identified using a multivariate Cox regression analysis (stepwise backward elimination with P < 0.05 for the remaining variables using the Akaike information selection criterion). These factors were further used to construct a prediction model in the derivation cohort. The β-coefficients from the final multivariable prediction model were used to create a point-scoring system for renal progression, as previously reported 18. Subsequently, a nomogram was constructed. We have provided some of the code for these analyses in Supplementary File 1. The model's discriminability was assessed by receiver operating characteristic (ROC) analysis. The calibration of prediction models was evaluated by visual inspection of calibration plots and the Brier score. We used decision curve analysis (DCA) to calibrate and evaluate the models, comparing predicted probabilities with actual probabilities. In addition, the nomograms were externally validated using a separate cohort of 102 patients with PMN from The Second Hospital of Longyan to assess their general applicability. The web-based application was developed using the "DynNom" package and "shinyapps" in R software. A two-sided P < 0.05 was defined as statistically significant.

## 3. Results

### 3.1 Baseline characteristics

**Figure 1:**
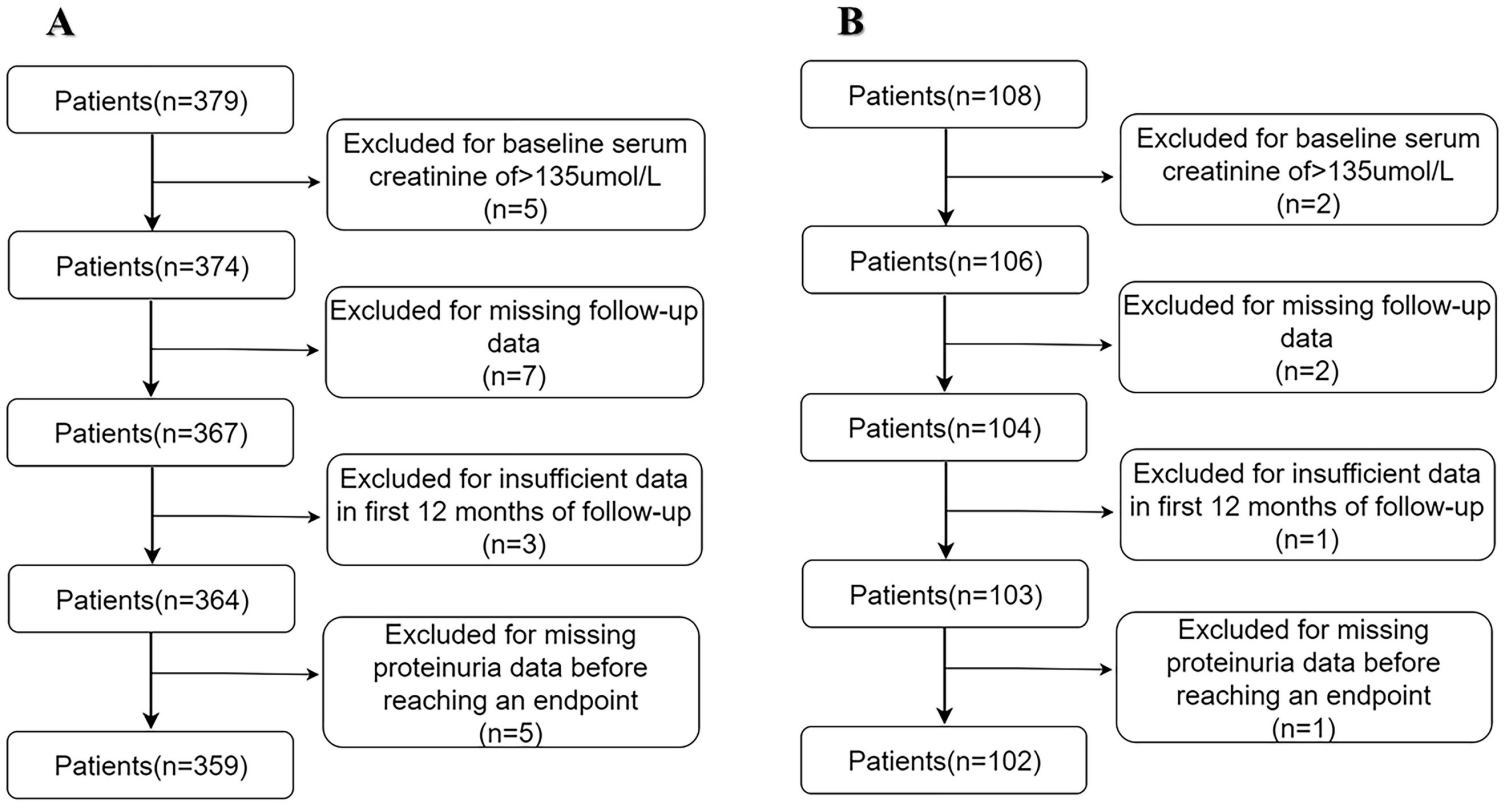
(A) Development cohort. (B) Validation cohort.

In total, 359 patients with PMN in the derivation cohort and 102 in the validation cohort were eligible for analysis (Figures 1A and 1 B). The demographic and clinical data are listed in Table 1 and Supplementary Table 1. After median follow-up durations of 37.60 ± 7.95 months and 29.94 ± 13.42 months, renal progression was observed in 18.3% and 21.6% of patients in the derivation and validation cohorts, respectively, with the majority being a reduction in eGFR (15.05% and 15.18% in the derivation and validation cohorts, respectively), and less frequently, ESRD (3.33% and 5.89% in the derivation and validation cohorts, respectively).

### 3.2 Risk factors of renal progression in PMN

In the derivation cohort, univariate analysis demonstrated that age, hyperuricemia, serum albumin, proteinuria, eGFR, and sPLA2R-ab were significantly correlated with renal progression in PMN patients (Table 2). Multivariate regression analysis revealed that hyperuricemia (HR=2.20, 95%CI 1.26 to 3.86), proteinuria (HR=2.16, 95%CI 1.47 to 3.18), significantly lower serum albumin (HR=2.34, 95%CI 1.51 to 3.68) and eGFR (HR=1.96, 95%CI 1.47 to 2.61), older age (HR=1.85, 95%CI 1.28 to 2.61), and higher levels of sPLA2R-ab (HR=2.08, 95%CI 1.43 to 3.18), were independent predictors of renal progression in PMN patients (Table 3).

### 3.3 Development and validation of a prediction model

**Figure 2:**
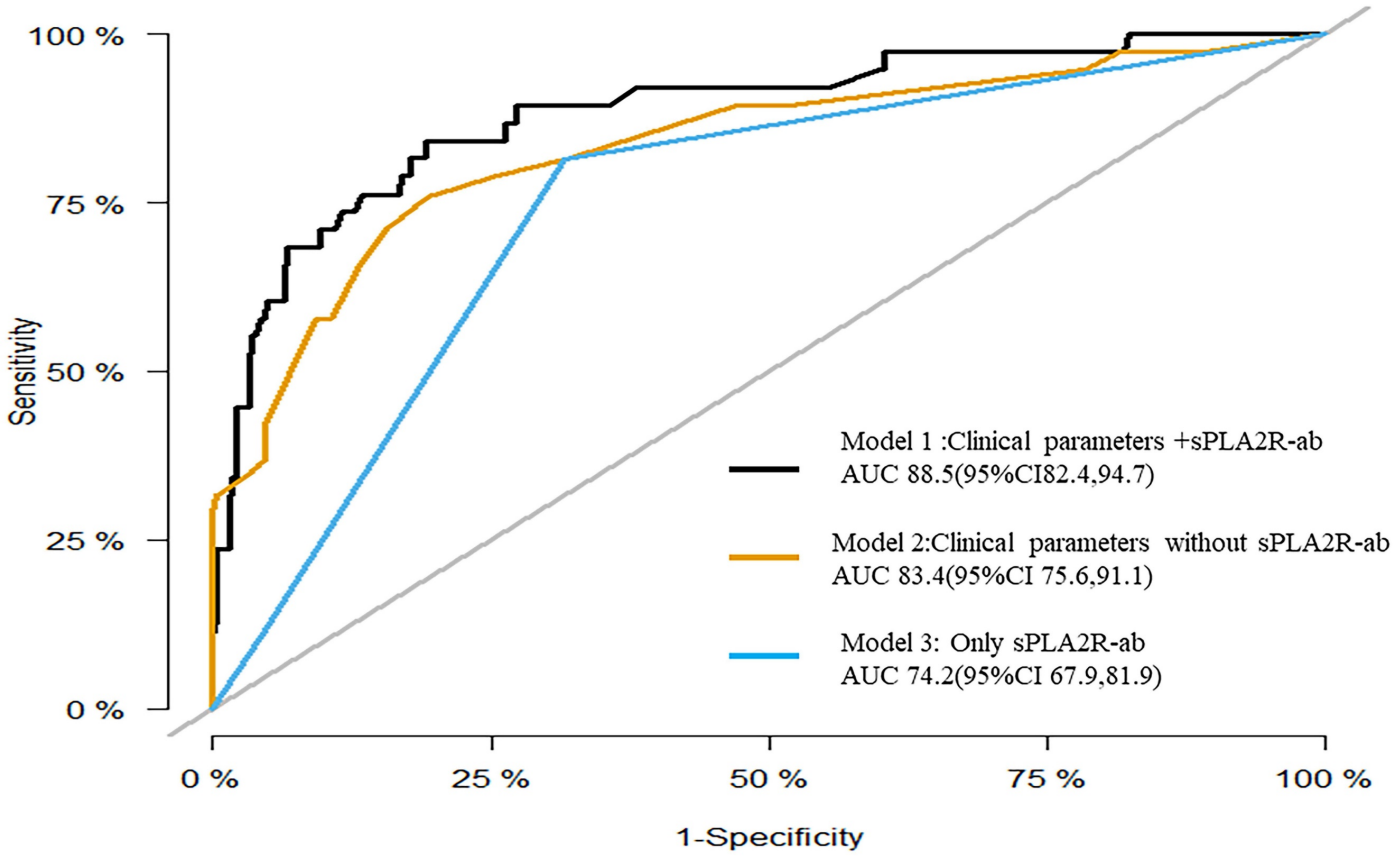
Receiver operating characteristic curves for the three models.

**Figure 3:**
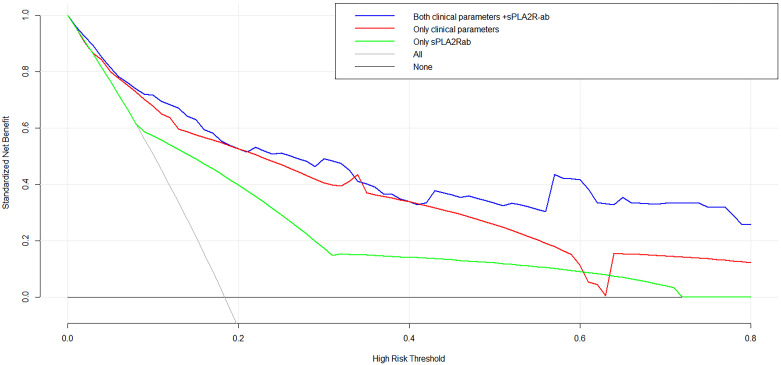
Decision curve analysis for the three models.

To assess the prognostic significance of sPLA2R-ab, now widely acknowledged as a potent clinical biomarker, we developed three distinctive models using notable risk factors associated with renal progression. These models include one that integrates sPLA2R-ab (Model 1), one that excludes sPLA2R-ab (Model 2), and one that solely relies on sPLA2R-ab (Model 3). As shown in the ROC analysis (Figure 2), Model 1 demonstrated excellent predictive power with an AUC of 0.89 (95% CI, 0.82 to 0.95), outperforming Model 2 (AUC of 0.84,95% CI, 0.76 to 0.92) and Model 3 (AUC of 0.74, 95% CI, 0.68 to 0.82). Similarly, compared to the other two models, Model 1 had the highest C-statistical value (both P < 0.01), establishing it as the best prediction model with acceptable calibration (Brier score = 0.062). In the DCA for models 1, 2, and 3, all models could be applied at threshold probabilities of 40%-60%. However, Model 1 offered superior net benefit at threshold probabilities of 40% to 80%, outperforming Model 2 and Model 3 (Figure 3). To further validate the predictive model's response to treatment, we performed a subgroup analysis using Model 1 on the corticosteroids and tacrolimus or cyclosporine treatment group. We found that Model 1 demonstrated good predictive ability for treatment response (AUC = 0.86, 95% CI: 0.73-0.98), as shown in Supplementary Figure 1.

In the validation cohort, renal progression was observed in 22 (21.6%) patients. Across the three risk categories, the validation cohort's rates were comparable to those observed in the derivation cohort. The prediction rule demonstrated good discrimination in the validation cohort (C-statistic = 72.7; 95%CI 58.2 to 87.3) and acceptable calibration (Brier score = 0.1554). The calibration plot is shown in Supplementary Figure 2. The predictive line (red line) overlaps well with the ideal line (black line), indicating that the predictive value is closely aligned with the actual value.

### 3.4 Development and validation of the risk scores

**Figure 4:**
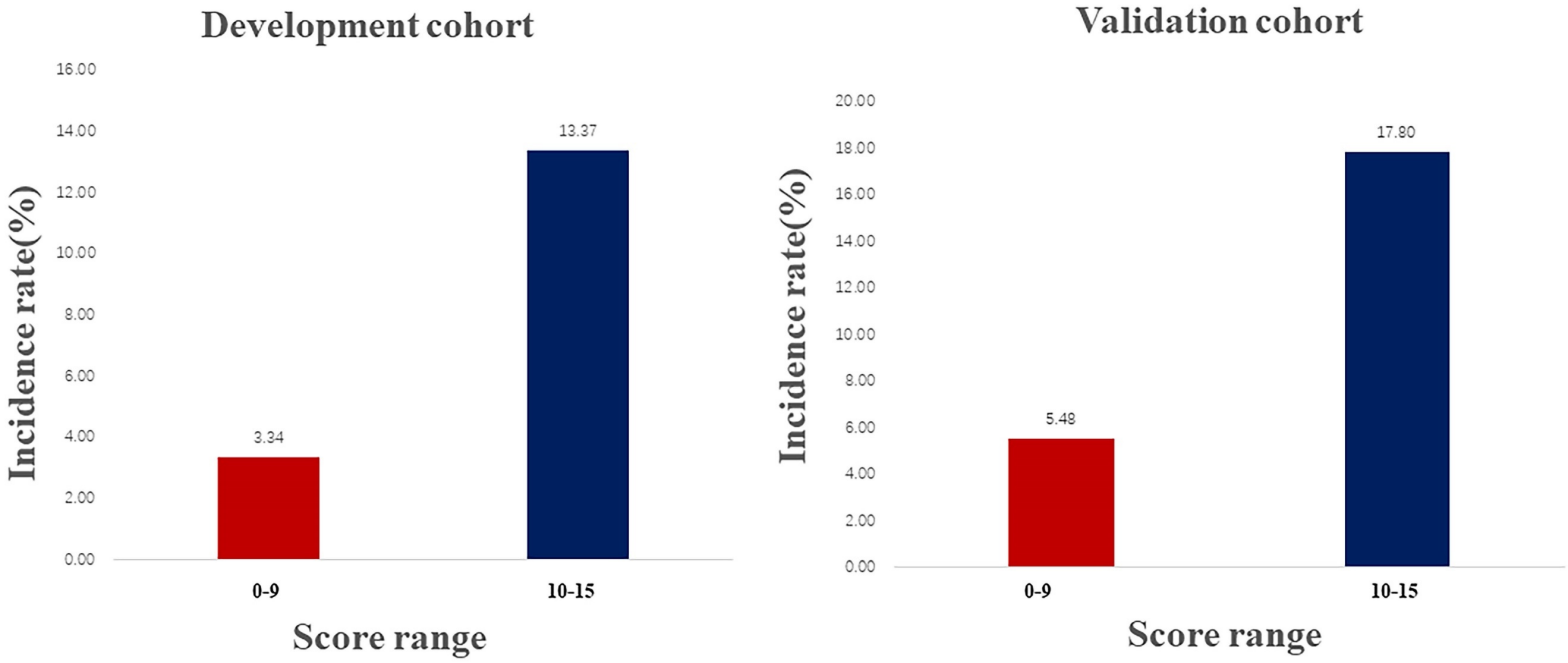
Frequency of renal progression in the development and validation cohorts across score ranges.

In the derivation cohort, renal function progression occurred in 18.3% of patients. To estimate the renal progression, a predictive score was developed based on the final model, following the method described by McMahon *et al.* 18. The incidence rate of the renal progression by risk score assigned to each predictive variable was listed in Table 4, increasing linearly with higher development cohort scores (Cochran Armitage Chi-square. P for trend < 0.001). According to the ROC analysis results (Supplementary Figure 3), The score ranged from 0 to 15, with a cutoff of 10. Thus, the total risk scores were divided into two groups: low (score 0-9), and high (score 10-15) risk. In both cohorts, a higher score was associated with a higher rate of renal progression: 3.34% and 5.48% for a score range of 0 to 9; and 13.37% and 17.90% for scores ranging from 10 to 15 (Figure 4).

### Development and internal validation of a web-based dynamic prediction model

**Figure 5:**
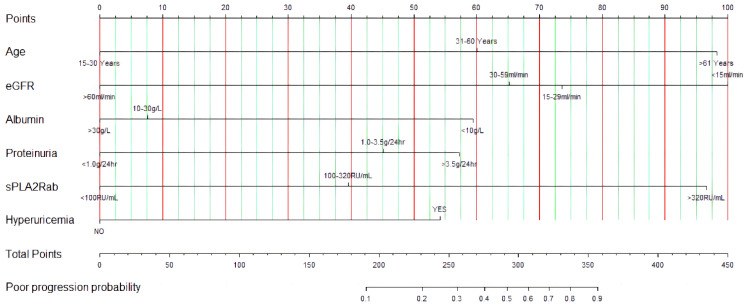
The predictive nomogram of renal progression for patients with PMN.

We developed a web-based dynamic nomogram for individualized estimation based on the optimized prediction model. As shown in Figure 5, the probability of renal progression for an individual could be determined by the total score of all variables, enabling a clinician to estimate the individual probability of renal progression. An online version of the prediction model was implemented in a web-based application available at http://imnpredictmodel1.shinyapps.io/dynnomapp.

**Figure 6:**
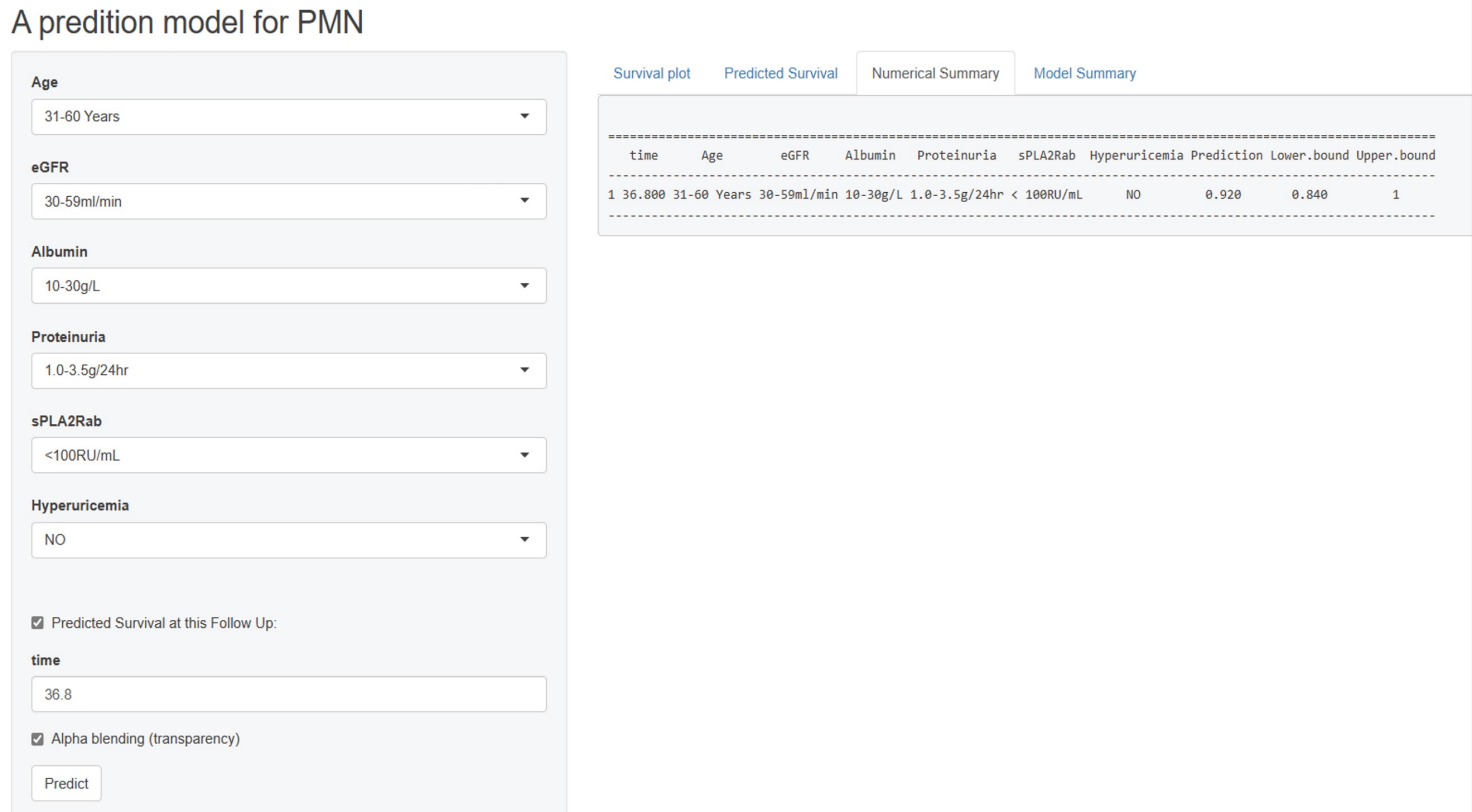
Screen shot of the web-based nomogram used to predict renal progression in patient with PMN (http://imnpredictmodel1.shinyapps.io/dynnomapp).

Figure 6 presents a screenshot of a renal progression nomogram from the web-based application. It depicts an example of a 45-year-old man diagnosed with PMN without significant medical history. His baseline eGFR is 50 ml/min/1.73m^2^, serum PLA2R antibody is 75 RU/mL, serum uric acid is 300 mmol/L, proteinuria is 2.5 g/24h, and his serum albumin is 29 g/L. Therefore, the predicted probability of renal progression over the following 36.8 months on the website would be 8%.

## Discussion

A valid tool for predicting the risk of renal function progression may help decision-making regarding treatment and follow-up strategy in PMN patients. Our study developed and validated a practical and simple prediction model for accurate stratification of the risk of renal progression in PMN patients. Additionally, our user-friendly web-based dynamic nomogram offers a tool for predicting the risk of renal progression in newly diagnosed PMN patients.

Age, proteinuria, and eGFR are well-established risk factors associated with poor prognosis in PMN patients 11, 13, 19. Additionally, serum albumin and hyperuricemia have been identified as indicators of disease severity and the risk for renal deterioration in PMN patients 4. Consistent with previous findings, our multivariate Cox analysis revealed that older age, hyperuricemia, lower serum albumin, heavier proteinuria, and lower eGFR were independently associated with renal progression in PMN patients. sPLA2R-ab is the primary pathogenic antibody in PMN, and its titer levels are associated with disease activity 14, 15. In 2021, the Kidney Disease Improving Global Outcomes (KDIGO) recommended that quantitative detection and regular monitoring of sPLA2R-ab for differential diagnosis and assessment of activity in PMN 16. The dynamic measurement of sPLA2R-ab has been widely recognized as a sensitive and specific prognostic biomarker to predict renal progression 6, 14. While previous studies have revealed the relationship between these parameters and PMN prognosis, most have focused on a single indicator. Evidence suggests that integrating multiple parameters for risk prediction can provide more accurate and comprehensive clinical value compared to a single variable 20, 21. The strength of our study lies in the integration of multiple key clinical parameters known to be associated with PMN prognosis into a web-based dynamic prediction model. This model incorporates multiple risk factors, including hyperuricemia, proteinuria, hypoalbuminemia, decreased eGFR, older age, and elevated sPLA2R antibody levels, enabling dynamic assessment of the risk of renal progression at different time points. Furthermore, the model demonstrates good discrimination and calibration, and has been validated in an independent cohort, supporting its reliability and generalizability. The web-based design makes it easy to operate, helping physicians quickly obtain personalized risk predictions for patients, providing strong support for clinical decision-making.

Despite the existence of several prognostic models 11-13, 15 for PMN, their applicability in clinical practice can be limited, as certain models include variables that are only applicable to specific types of PMN, or they may not align with the actual prognosis observed in clinical practice. Additionally, PMN patients at different disease stages exhibit varying responses to treatment and have varying prognoses over time. Herein, our dynamic predictive model, which considers patient specificity, incorporates various risk factor changes, screens for the impact of specific immunosuppressive treatment, and holds the potential to accurately delineate correlations between disease severity and prognosis on an individual basis. This dynamic model allows clinicians to use all available information (baseline and follow-up) to accurately detect disease progression, effortlessly update patient prognosis, and perform real-time risk assessment, thereby yielding significant clinical benefits. By incorporating treatment response prediction, our model provides a comprehensive tool for personalized management of PMN patients, enabling clinicians to make informed decisions regarding treatment strategies and monitoring plans based on individual patient characteristics and disease progression.

We further developed a user-friendly web-based dynamic prediction model for individualized estimation of the risk of renal progression. Compared to a previous dynamic prediction model 13, 15 that roughly calculated an approximation, our model offers a simple interface, allowing clinicians to input patient information and obtain real-time predictions, facilitating risk stratification and prognostication. For example, a patient with an 8% probability of renal progression despite low eGFR at diagnosis may benefit from observation or conservative treatment, while immunosuppressive agents should be withheld to avoid adverse effects. The web-based platform enhances accessibility and usability, streamlining data input and interpretation, thereby improving clinical decision-making and optimizing patient care in PMN management. By providing a convenient and reliable tool for individualized risk assessment, our model has the potential to facilitate its adoption in clinical settings.

Nevertheless, several limitations need to be mentioned. First, this study may be subjected to potential selection bias due to the retrospective study design. Second, our risk prediction models were developed and validated based on a cohort of Chinese patients; its applicability to other ethnicities such as Caucasian or Black MN patients remains to be further validated. Third, the median follow-up for our study was only 37.6 months; caution should be taken when applying this model to estimate long-term renal outcomes. Lastly, more recently described target antigens, such as thrombospondin type1 domain-containing 7A, semaphoring 3B, neural epidermal growth factor-like 1 protein, and protocadherin 7 22-24, were not evaluated in the present study.

## Conclusions

In conclusion, our study developed and externally validated a user-friendly web-based dynamic prediction model to estimate individual risk of renal progress in PMN patients. This tool may be helpful in decision-making regarding personalized treatment and surveillance strategies for PMN patients.

## Supplementary Material

Supplementary information, figures and tables.

## Figures and Tables

**Table 1:** Baseline characteristics of the derivation cohort (n=359)

Characteristics	All
Age, mean (range), years	54 (45 - 63)
Female, n (%)	156 (43.5%)
Diabetes mellitus, n (%)	71 (19.8%)
MAP, mean (range), mmHg	96 (89 -107)
BMI, mean ± SD, kg/m^2^	23.11 ± 5.16
Laboratory	
eGFR, mean ± SD, ml/min/1.73m^2^	94.73 ± 23.15
Serum uric acid, median (range), umol/L	376.65 (148 - 764)
Serum cholesterol, mean ± SD, mmol/L	8.14 ± 2.67
Serum albumin, mean ± SD, g/L	25.48 ± 6.21
Serum PLA2R antibody mean (IQR), RU/mL	85.3 (29.3, 167.9)
Pathology, n (%)	
LM-stages I and II	187 (52.1%)
LM-stages III	118 (32.9%)
LM-stages IV	33 (9.2%)
LM-stages V	21 (5.8%)
LM- ≥ 50% tubulointerstitial lesions	14 (3.95%)
IF-PLA2R positive staining	234 (65.13%)
IF-IgG1 positive	59 (16.4%)
IF-IgG4 positive	303 (84.32%)
Immunosuppressants (n, %)	
None (n, %)	78 (21.72%)
CTX (n, %)	87 (24.23%)
CNI (n, %)	102 (28.41%)
RTX (n, %)	28 (7.80%)
Other (n, %)	74 (20.61%)
ACEIs (n, %)	125 (34.81%)
ARBs (n, %)	212 (59.05%
Outcomes, n (%)	
Renal function progression	66 (18.3%)
30% reduction in eGFR, n (%)	54 (15.05%)
ESRD, n (%)	12 (3.33%)
Follow up, mean ± SD, months	37.60 ± 7.95

SD: standard deviation, IQR: interquartile range, MAP: mean arterial pressure, BMI: body mass index, eGFR: estimated glomerular filtration rate, PLA2R: phospholipase A2 receptor, LM: light microscopic, IF: immunofluorescence, ESRD: end-stage renal disease

**Table 2:** Univariate analysis of the association between variables and renal progression in the derivation cohort

Variable	Patients (n)	Events (n)	HR	95% CI	*P* value
Age, years					
15-35	120	5	Reference		
36-60	153	25	2.85	1.00-8.10	0.05
>61	86	30	4.52	1.68-12.14	0.003
Gender					
Female	156	30	Reference		
Male	203	36	1.104	0.65-1.89	0.779
Hyperuricemia					
No	195	22	Reference		
Yes	164	44	3.58	1.87-6.88	0
Hypercholesterolemia					
No	112	22	Reference		
Yes	247	44	0.77	0.42-1.44	0.413
Serum albumin, g/L					
>30	97	5	Reference		
10-30	202	34	1.41	0.50-3.96	0.521
<10	60	27	3.91	1.24-12.35	0.02
Proteinuria, g/24hr					
<1.0	178	12	Reference		
1.0-3.5	130	27	2.62	1.15-5.99	0.022
>3.5	51	27	4.17	1.83-9.52	0.001
eGFR, ml/min/1.73m^2^					
>60	232	16	Reference		
30-59	70	19	2.7	1.19-6.05	0.017
15-29	52	27	5.74	2.70-12.16	0
<15	5	4	9.2	2.32-36.46	0.002
Pathology					
LM-stages I and II (%)	187	25	Reference		
LM-stages III (%)	118	28	2.01	1.10-3.67	0.024
LM-stages IV (%)	33	9	3.14	1.34-7.36	0.008
LM-stages V (%)	21	4	0.63	0.18-2.22	0.473
sPLA2R-ab (RU/L)					
Negative	82	9	Reference		
Low titer	147	10	0.25	0.09-0.71	0
Middle titer	115	35	1.12	0.47-2.69	0
High titer	15	12	2.27	0.72-7	0.49

CI: confidence interval, HR: hazard ratio, eGFR: estimated glomerular filtration rate, sPLA2R-ab: serum phospholipase A2 receptor antibody Hypercholesterolemia is diagnosed by baseline serum cholesterol > 5.72mmol/L. Hyperuricemia diagnosed by baseline serum uric acid > 360umo/L in females and > 420umol/L in males.

**Table 3:** Multivariate COX analysis of predictors of the combined endpoint in the development cohort

Variables	Model coefficient	HR	95% CI	*P* value
Age,years	0.61	1.85	1.28-2.67	0.001
eGFR, ml/min/1.73m^2^	0.67	1.96	1.47-2.61	0
Serum albumin, g/L	0.85	2.34	1.51-3.63	0
Proteinuria, g/24hr	0.77	2.16	1.47-3.18	0
sPLA2R-ab (RU/L)	0.73	2.08	1.43-3.18	0.001
Hyperuricemia	0.79	2.20	1.26-3.86	0.006

eGFR: estimated glomerular filtration rate, sPLA2R-ab: serum phospholipase A2 receptor antibody, HR: hazard ratio, CI: confidence interval

**Table 4:** Multivariate COX analysis of predictors of renal progression with Score generation in the derivation cohort

Variables	No. (%)	β Estimate	HR	95%CI			P value	Score Assigned
Age, 15-30years	120 (33.4)	Ref	Ref	Ref			Ref	0
Age, 31-60years	153 (42.6)	1.117	3.056	1.121	to	8.335	0.029	2
Age, >60years	86 (24)	1.183	6.217	2.331	to	16.578	0.002	3
eGFR, >60ml/min.1.73m^2^	232 (64.6)	Ref	Ref	Ref			Ref	0
eGFR, 30-59ml/min.1.73m^2^	70 (19.5)	1.214	3.365	1.607	to	7.047	0.000	2
eGFR, >15-29ml/min.1.73m^2^	52 (14.5)	1.367	3.930	2.029	to	7.612	0.000	3
eGFR, <15ml/min.1.73m^2^	5 (1.4)	1.858	6.410	1.900	to	21.626	0.000	4
Serum albumin, >30g/L	97 (27)	Ref	Ref	Ref			Ref	0
Serum albumin, 10-29g/L	202 (56.3)	0.142	1.153	0.407	to	3.260	0.789	1
Serum albumin, <30g/L	60 (16.7)	1.105	3.020	0.964	to	9.464	0.050	3
Proteinuria, <1.0 g/24hr	178 (49.6)	Ref	Ref	Ref			Ref	0
Proteinuria, 1.0-3.5 g/24hr	130 (36.2)	0.840	2.316	0.954	to	5.627	0.063	1
Proteinuria, >3.5 g/24hr	51 (14.2)	1.065	2.902	1.117	to	7.541	0.029	3
sPLA2R-ab, < 100RU/L	229 (63.8)	Ref	Ref	Ref			Ref	0
sPLA2R-ab, 100-320RU/L	114 (31.7)	0.735	2.086	1.086	to	4.008	0.028	2
sPLA2R-ab, > 320RU/L	16 (4.5)	1.794	6.034	2.526	to	14.410	0.000	3
Hyperuricemia	164 (45.7)	1.008	2.741	1.521		4.940	0.007	1

eGFR: estimated glomerular filtration rate, sPLA2R-ab: serum phospholipase A2 receptor antibody
