# Supplementary material for: A Dynamic Prediction Model for Renal Progression in Primary Membranous Nephropathy
Source: Int J Med Sci. 2024 May 13;21(7):1292–301. doi: 10.7150/ijms.95321 (PMC11134588; doi:10.7150/ijms.95321)
Supplement: Supplementary file 1 — Supplementary information, figures and tables. [file ijmsv21p1292s1.pdf]

Supplementary File 1

```
#read data
data=read.spss("C:/Users/Desktop/data.sav",use.value.labels = F,to.data.frame = T)
head(data)

# exclude all missing values (complete cases analaysis)

data <- na.omit(data)
View(data)

# define outcome and predictors

Outcome <- "Surv(time,Status)"
CandidateVariables <- c("Age","DM","MAP","BMI","Serum uric acid","Serum albumin","pathology","eGFR","Albumin","Proteinuria","sPLA2Rab","gender","HBP","Hypercholesterolemia","IS treatment")

# create a formula
Formula <- formula(paste(paste(Outcome,"~", collapse=" "),
                         paste(CandidateVariables, collapse=" + ")))


# fit a model with all candidate varaibles
model.full <- coxph(Formula, data=data,x=TRUE)

summary(model.full)

# stepwise selection

model.step <- stepAIC(model.full, direction="both")

summary(model.step)

# evaluate model at time = 12

time <- 12

# in training set

plotROC(Score(list("model.step"=model.full),Surv(time,Status)~1,data=data,times=time,
plots="roc",metrics=c("AUC")))

plotCalibration(Score(list("model.step"=model.step),Surv(time,Status)~1,data=data,times
=time, plots="cal",metrics=c("AUC","Brier")),cens.method="local")
# bootstrap validation (it is called cross validation, but acturally it is bootstrap validation)
```

```
plotCalibration(Score(list("model.step"=model.step),Surv(time,Status)~1,data=data,times
=time,
plots="cal",metrics=c("AUC","Brier"),split.method="bootcv",B=100,N=nrow(data)),cens.
method="local")


c_harrell_apparent<-(cph(Surv(time,Status)~p_lp,data=data,x=TRUE,y=TRUE)$stats["D
xy"]+1)/2
c_harrell_apparent

c_time_apparent<-Score(list("model.step"=model.step),Surv(time,Status)~1,data=data,
times=time, plots="cal",metrics=c("AUC"))$AUC$score$AUC
c_time_apparent

brier_apparent<-Score(list("model.step"=model.step),Surv(time,Status)~1,data=data,time
s=time, plots="cal",metrics=c("Brier"))$Brier$score$Brier[-1]
brier_apparent
```

Supplementary Table 1. Baseline characteristics of the Validation cohort (n=102)

**Characteristic**	**All**
Age, mean (range), years	54 (45 - 66)
Female, n (%)	44(44.1%)
Diabetes mellitus, n (%)	38(37.3%)
MAP, mean (range), mmHg	96(84 - 107)
BMI, mean ± SD, kg/m^2	23.91 ± 6.36
**Laboratory**	
eGFR, mean ± SD, ml/min/1.73m^2	78.79 ± 29.70
Serum uric acid, median (range), umol/L	367(296.8 - 450)
Serum cholesterol, mean ± SD, mmol/L	7.99± 2.65
Serum albumin, mean ± SD, g/L	25.22 ± 8.42
Serum PLA2R antibody mean (IQR), RU/mL	112（24.81，250）
**Pathology**, n (%)	
LM-stages I and II	52 (50.9%)
LM-stages III	36 (35.3%)
LM-stages IV	10 (9.8%)
LM-stages V	6 (5.9%)
LM- ≥ 50% tubulointerstitial lesions	3 (2.9%)
IF-PLA2R positive staining	62 (60.78%)
IF-IgG1 positive	73 (71.5%)
IF-IgG4 positive	84 (82.35%)
**Immunosuppressants (n, %)**	
None (n, %)	22(21.56%)
CTX(n, %)	23(22.55%)
CNI(n, %)	35(34.31%)
RTX(n, %)	18(17.64%)
Other(n, %)	4(3.92%)
ACEIs (n, %)	45 (44.12%)
ARBs (n, %)	52 (50.98%)
**Outcomes**, n (%)	
Renal function progression	22 (21.6%)
30% reduction in eGFR, n (%)	16（15.18%）
ESRD, n (%)	6 (5.89 %)
Follow up, mean ± SD, months	29.94 ± 13.42

SD: standard deviation, IQR: interquartile range, MAP: mean arterial pressure, BMI: body mass index, eGFR: estimated glomerular filtration rate, PLA2R: phospholipase A2 receptor, LM: light microscopic, IF: immunofluorescence, ESRD: end-stage renal disease

SupplementaryTable 2 Validation three prediton models in the development cohort

**Prediction performance**		**Development cohort**	**Validation cohort**
C-statistic(95%CI)	Model 1	0.89 (95% CI, 0.82 to 0.95)	0.73(95%CI,0.58 to 0.87)
	Model 2	0.84 (95% CI, 0.76 to 0.92)	0.69(95%CI,0.42 to 0.77)
	Model 3	0.74 (95% CI, 0.68 to 0.82)	0.61(95%CI,0.54 to 0.84)
Brier score	Model 1	0.062	0.1554
	Model 2	0.077	0.209
	Model 3	0.117	0.283

Model1,clinical parameters with sPLA2R-ab; Model 2, clinical parameters without sPLA2R-ab; Model3,only with sPLA2R-ab.

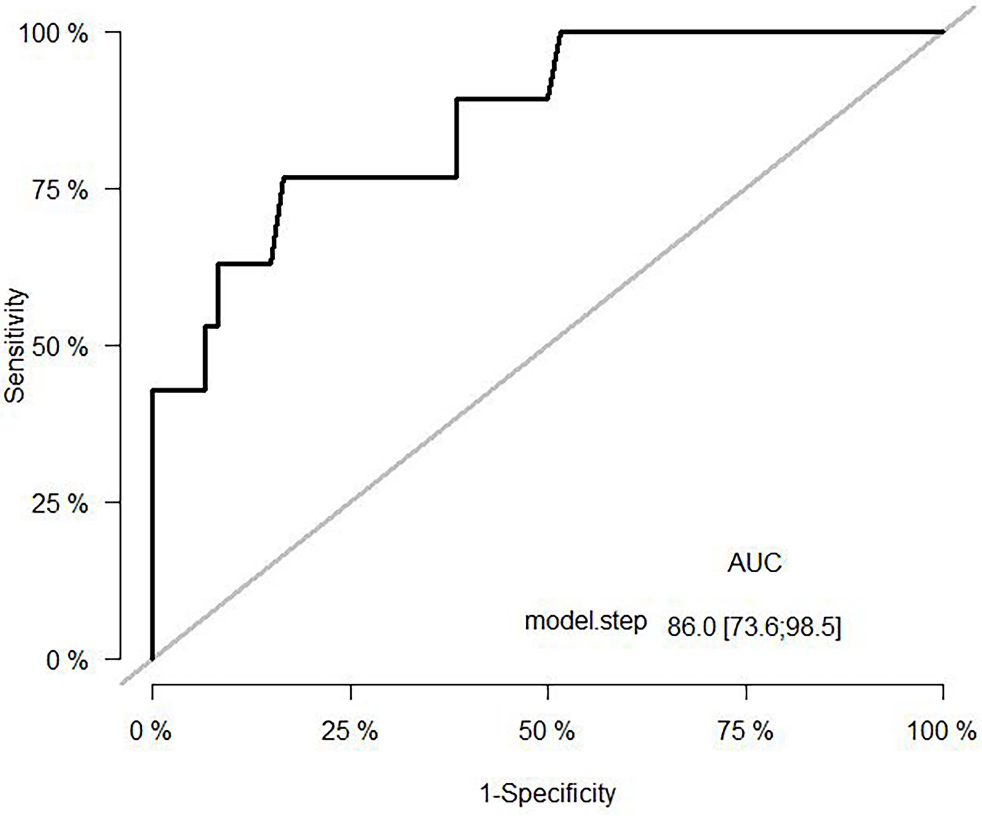

100 %
75 %
50 %
25 %
0 %
Sensitivity
0 %
25 %
50 %
75 %
100 %
1-Specificity
AUC
model.step
86.0 [73.6;98.5]

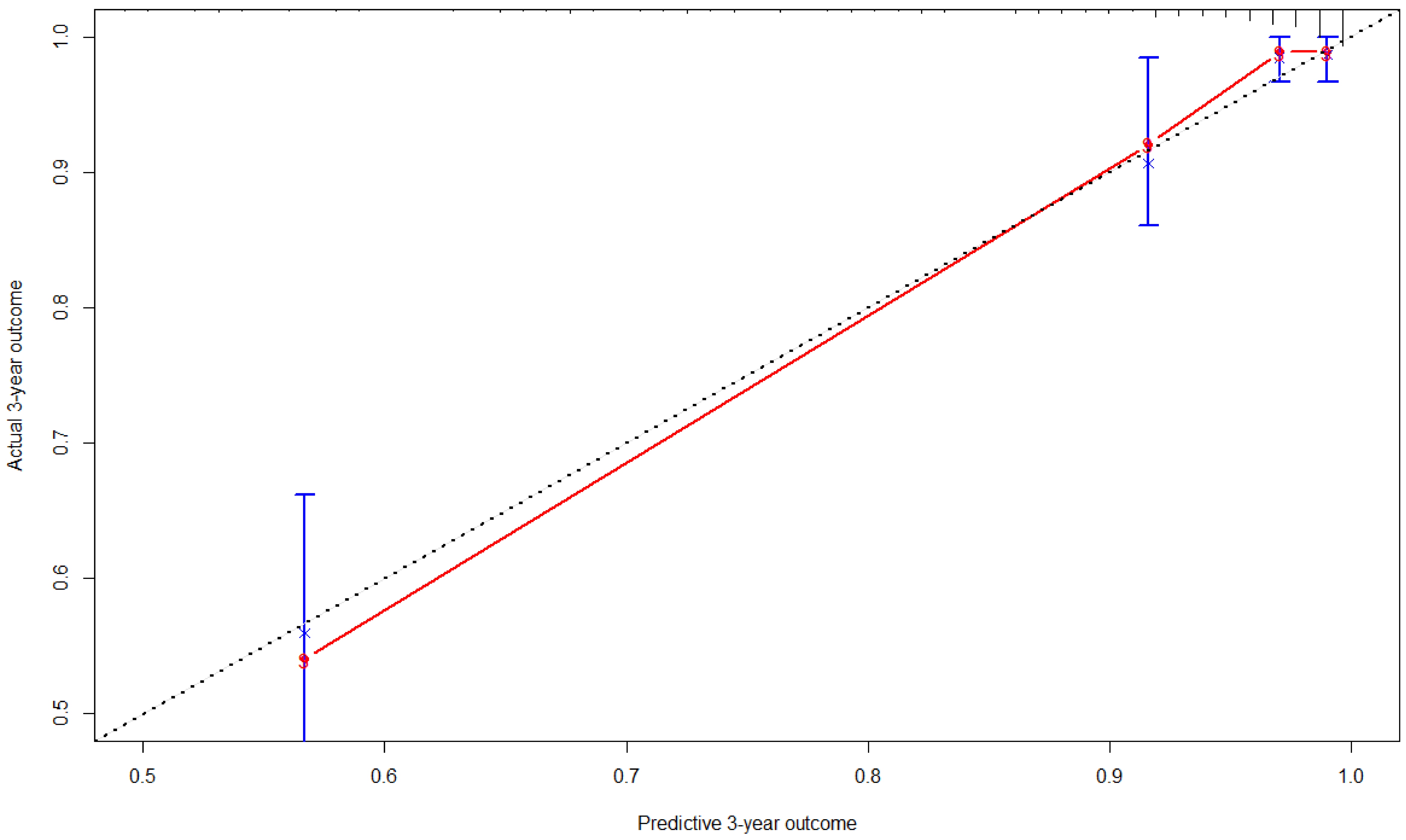

Actual 3-year outcome
1.0
0.9
0.8
0.7
0.6
0.5
0.5
0.6
0.7
0.8
0.9
1.0
Predictive 3-year outcome

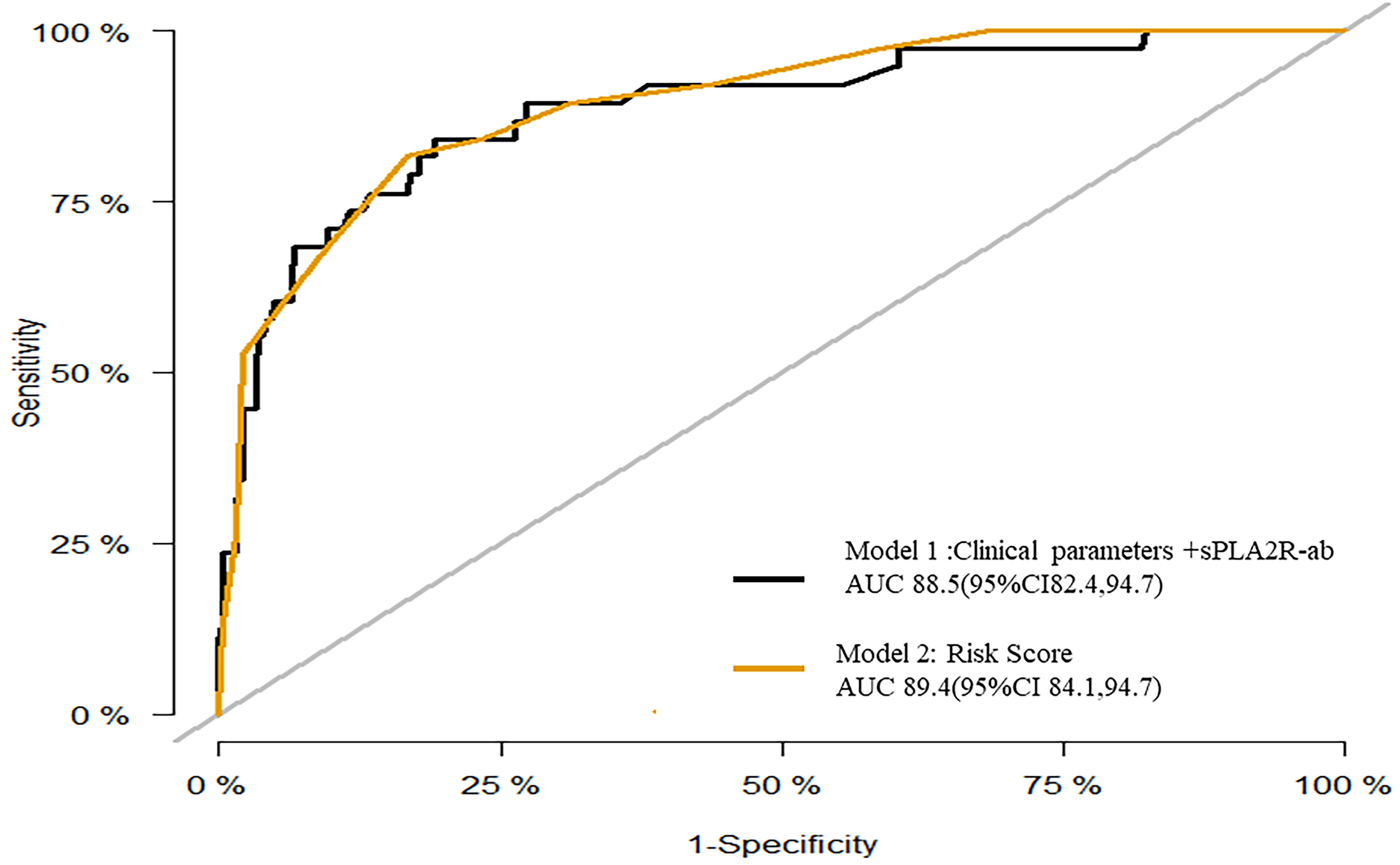
100 %
75 %
50 %
25 %
0 %
Sensitivity
0 %
25 %
50 %
75 %
100 %
1-Specificity
Model 1 :Clinical parameters +sPLA2R-ab
AUC 88.5(95%CI82.4,94.7)
Model 2: Risk Score
AUC 89.4(95%CI 84.1,94.7)
